# Unruptured ovarian ectopic pregnancy: Two case reports and literature review

**DOI:** 10.3389/fphys.2022.1036365

**Published:** 2022-10-25

**Authors:** Fang Ren, Gang Liu, Tifang Wang, Meijun Li, Zhiqiang Guo

**Affiliations:** ^1^ Department of Obstetrics and Gynecology, Shengjing Hospital of China Medical University, Shenyang, China; ^2^ Department of Urology, Shengjing Hospital of China Medical University, Shenyang, China

**Keywords:** ovarian pregnancy, diagnosis, treatment, case report, review

## Abstract

In clinical practice, ovarian pregnancy is extremely rare and is always found to be ruptured. A definitive diagnosis for ruptured ovarian pregnancy is difficult to obtain. We present two cases of unruptured ovarian pregnancies detected during laparoscopy and review existing literature to better understand the clinical characteristics of ectopic pregnancies in this rare site. Intrauterine devices, assisted reproductive technology, and intrauterine operations are all high-risk factors in ovarian pregnancy. Moreover, menopause, abdominal pain, and vaginal bleeding are clinical manifestations. Ovarian pregnancy can be diagnosed using serum hCG, transvaginal ultrasound, and magnetic resonance imaging. Laparoscopy is the treatment of choice for ovarian pregnancy. It is recommended that the intact gestational sac be excised and the ovarian function be protected to the greatest extent possible during the operation. More definitive diagnosis of ovarian pregnancy must be reported in order to gain a better understanding of ovarian pregnancy.

## Background

The most common site of ectopic pregnancy (EP) is the fallopian tube. Ovarian pregnancies are a rare occurrence, accounting for 0.5%–3.0% of all EPs (Ghasemi et al., 2014). Its incidence may be increasing because of improved diagnostic techniques and assisted reproductive technology (Abidi et al., 2017).

Given that the surface cortex of the ovarian pregnancy tissue is thin and lacks elasticity, ovarian pregnancies were usually found to be ruptured, and an intact gestational sac on the ovary is rarely seen in clinical practice. Clinically, an ovarian pregnancy is often diagnosed on the basis of the normal shape of the bilateral fallopian tubes, bleeding lesions on the ovarian surface, and visible or invisible pregnancy tissue in the pelvic cavity. However, given that this situation is difficult to distinguish from ovarian corpus luteum rupture combined with tubal pregnancy abortion, a definite diagnosis for ruptured ovarian pregnancy is difficult to obtain.

We present two cases of unruptured ovarian pregnancy during laparoscopy and review existing literature to better understand the clinical characteristics of ectopic pregnancy in this rare site.

## Case presentation

### Case 1

The patient was a 31-year-old female (gravida 0, para 0) with regular menstruation. She underwent *in vitro* fertilization and embryo transfer (IVF-ET), through which one embryo was implanted. On routine examination 24 days after transplantation, serum human chorionic gonadotropin (hCG) was 9448 IU/L. Color Doppler ultrasonic showed an empty uterus and the left ovary contained a mass that was 2.2 × 2.0 × 1.8 cm^3^ in size. The boundary of the mass was clear, the periphery presented a medium echo, and the center was liquid. CDFI further detected circular blood flow signals in the periphery of the mass. Ultrasound diagnosis was left ovarian mass (corpus luteum?).

**FIGURE 1:**
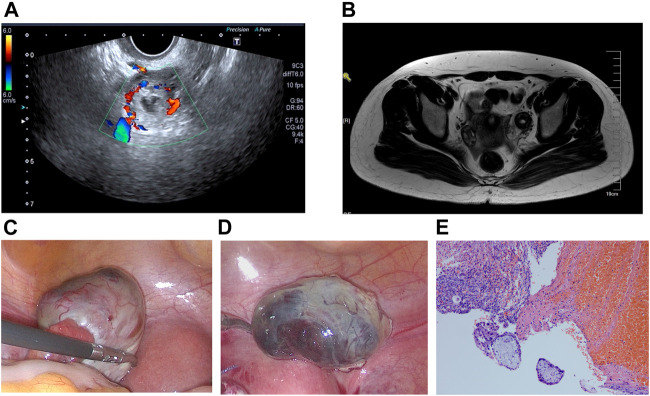
**(A)** Transvaginal ultrasonography revealed an echogenic mass in the left ovary. **(B)** MRI diagnosis revealed a left ovarian mass. **(C)** Ovarian pregnancy was detected using a laparoscope. **(D)** Ovarian pregnancy lesion was completely removed. **(E)** Postoperative pathological analysis confirmed left ovarian gestation, revealing both chorionic villi and trophoblasts.

The mass in the ovary was not excluded from the physiological corpus luteum, and the embryo is valuable; thus, the patient required conservative treatment and was dynamically monitored with hCG and ultrasound. During the monitoring, the hCG value rises slowly, and the mass gradually increases (Figure 1A). Further pelvic magnetic resonance imaging (MRI) showed a quasi-circular mixed-signal nodule of ×2.7 2.4 cm^2^ in size with a complete capsule and a thin wall at the front edge of the left ovary. T1 imaging was dominated by a low signal with a few arc-shaped high signals at the lower edge, whereas T2 imaging showed mixed signals dominated by a high signal, and a small, nodule-like low-signal shadow could be seen scattered at the edge (Figure 1B).

Considering the continuously increasing hCG and the presence of the ovarian mass, the high possibility of ovarian pregnancy was inferred. Following the patient’s consent, laparoscopic exploration was indicated. Observations during the operation included a normal-sized uterus, smooth and normal fallopian tubes, normal right ovary and a purple-and-blue mass on the surface of the left ovary measuring 4.0 × 2.5 cm^2^ with an intact capsule partially fused with the ovarian cortex, and abundant surface blood vessels (Figure 1C). Laparoscopic resection of the ovarian pregnancy lesion was performed through laparoscopic scissors. The gestational sac of the ovarian pregnancy was intact upon removal (Figure 1D), and the ovary was sutured and repaired. Postoperative pathological analysis confirmed left ovarian gestation, demonstrating both chorionic villi and trophoblasts (Figure 1E). The patient had an uneventful postoperative period and was discharged without complications. After the operation, serum hCG gradually returned to normal levels. Eight months after operation, the patient underwent a second embryo transfer and is now 32 weeks pregnant.

### Case 2

**FIGURE 2:**
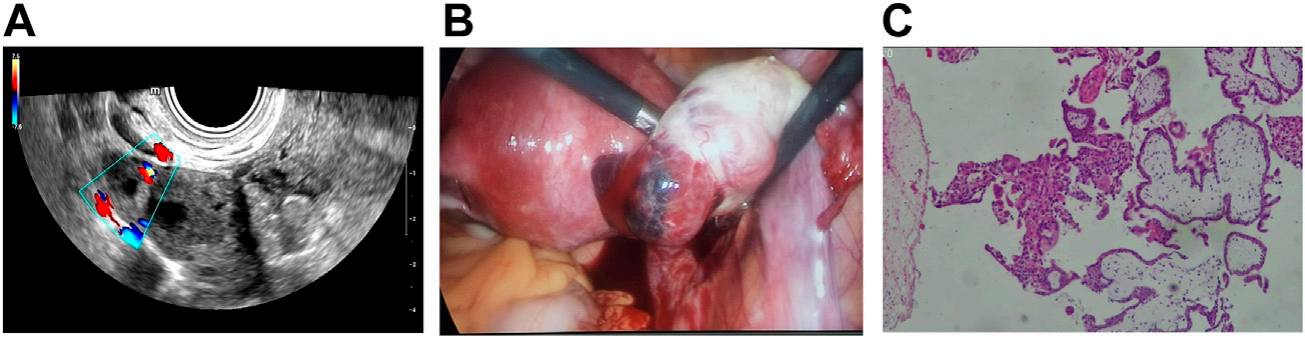
**(A)** Transvaginal ultrasonography revealed an echogenic mass in the right ovary. **(B)** Ovarian pregnancy was detected using a laparoscope. **(C)** Postoperative pathological analysis confirmed right ovarian gestation, revealing both chorionic villi and trophoblasts.

The patient was a 38-year-old female (gravida 4, para 2) with regular menstruation. The patient’s surgical history included an appendectomy, two deliveries *via* cesarean section, and hysteroscopic curettage (pathological diagnosis was proliferative endometrium). She presented to the emergency department of our hospital 44 days after menopause with mainly complaining of pain in the right lower abdomen for 5 days. Her serum hCG was 6623 IU/L. The ultrasound revealed an empty uterus and 1.4 × 1.2 × 1.1 cm^3^ mass next to the right ovary, which was irregular in shape and slightly hyperechoic on the inside with a 0.7 × 0.4 × 0.4 cm^3^ liquid area in the center. CDFI could detect blood flow signals in the surrounding area (Figure 2A).

On the second day of admission, serum hCG levels increased, and the diagnosis of ultrasonic consultation was consistent with the admission ultrasonic report. Given the high possibility of an ectopic pregnancy and the increasing level of hCG, we performed an exploratory laparoscopy for the patient. During the procedure, a normal-sized uterus, smooth and normal fallopian tubes, normal left ovary, and purple-and-blue mass measuring 2.0 × 1.5 cm^2^ on the surface of the right ovary with an intact capsule (Figure 2B) were observed. Laparoscopic resection of the gestational sac was performed, and the ovary was repaired. Postoperative pathological analysis confirmed right ovarian gestation (Figure 2C). The patient’s postoperative period was uneventful, and serum hCG levels gradually returned to normal. The patient had no complications and was still being monitored 7 months after the operation.

## Discussion and conclusion

### High-risk factors

Ovarian pregnancy is a rare type of EP, and the pathogenesis remains unclear. Traditional risk factors for tubal EP (e.g., fallopian tube or pelvic inflammation) are not relevant in ovarian pregnancy. Presumed risk factors for ovarian pregnancy include prior use of the intrauterine device (IUD), assisted reproductive techniques, concurrent endometriosis, pelvic adhesions, and intrauterine surgery (Ghasemi et al., 2014; Marcus et al., 1993; Ciortea et al., 2013).

The relationship between IUD and ovarian pregnancy is the most established and recognized. IUD use ranges from 57% to 90% of patients with a primary ovarian pregnancy (Comstock et al., 2004; Ghi et al., 2005; Raziel et al., 1990; Herbertsson et al., 1987; Cabero et al., 1989). A 10-year study of a large series of 1800 EPs indicated the current IUD use as the only risk factor associated with the site of EP in distal EP and ovarian pregnancies (Sergent et al., 2002). IUD use may alter tubal motility, thereby facilitating ovarian implantation.

In recent years, increasing cases of ovarian pregnancies have also been described following IVF-ET (Einenkel et al., 2000; Dursun et al., 2008; Selo-Ojeme et al., 2002). A few reasons have been considered (Paltieli et al., 2000; Zhu et al., 2014; Rizk et al., 1991). First, the application of ovulation-stimulating drugs can induce increased sex hormone secretion by the ovaries, thereby increasing the contractile sensitivity of uterine smooth muscle and interfering with the fallopian tube function. After embryo transfer, the zygote moves back into the fallopian tube and implants itself in the ovary. Second, the embryo may also become implanted in the ovary through a breach after ovulation or the needle path after egg retrieval. Third, the large volume of the culture medium injected into the uterus during transplantation, high injection pressure, and surgery-induced uterine contraction may cause an ovarian pregnancy. Fourth, the number of IVF-ET embryo and blastocyst transfers has also been associated with the occurrence of ovarian pregnancy. Case 1 confirms some of these possible causes as the patient had undergone IVF-ET and received gonadotropins or clomiphene citrate treatment.

Intrauterine surgery may alter the environment for intrauterine embryo implantation, increasing the likelihood of an ectopic pregnancy. The patient in case 2 underwent hysteroscopic curettage a year ago due to abnormal uterine bleeding. Herein, the history of intrauterine surgery may be the primary cause of the ovarian pregnancy.

In addition, concurrent endometriosis and pelvic adhesions can block ovulation, forcing the egg cell to stay in the ruptured follicle and develop in the ovary. Some scholars believe that the irregular maturation of follicles during ovulation could also cause ovarian pregnancy (Suikkari et al., 2007).

### Diagnostic criteria and classification of ovarian pregnancy

Diagnostic criteria for ovarian pregnancy described by Spiegelberg (1973) include ovarian attachment to the uterus *via* the ovarian ligament, location of the gestational sac in or around the ovary, intact fallopian tube but with its fimbria and separated from the ovary, and ovarian tissue in the specimen on histological analysis.

Since the surface cortex of the ovarian pregnancy tissue is thin and lacks elasticity, the ovarian pregnancy was always found to be ruptured. Clinically, the discovery of intact fallopian tubes, bleeding lesions on the ovarian surface, and visible or invisible pregnancy tissue in the pelvic cavity is frequently used to diagnose ruptured ovarian pregnancies. However, this situation is difficult to distinguish from ovarian corpus luteum rupture with tubal pregnancy abortion. The two cases of unruptured ovarian pregnancies presented here may aid in our understanding of the pathophysiological and clinical characteristics of ovarian pregnancies.

Ovarian pregnancy can be classified as intrafollicular or extrafollicular ovarian pregnancy based on etiology (Tan et al., 1968). In the intrafollicular type, the oocyte is not discharged from the follicle during ovulation. Subsequently, the sperm enters through the ruptured opening and initiates fertilization in the follicle. The extrafollicular type occurs when an oocyte has been released from the follicle but becomes implanted on the ovarian surface after fertilization. Nakagawa et al. (2004) categorized ovarian pregnancies into mass formation type or outward development type according to surgical findings. These two developmental patterns appear to correspond to the clinical intrafollicular and extrafollicular phenotypes.

### Clinical manifestation

The clinical manifestations of ovarian pregnancy are similar to those of tubal EP, which include menopause, abdominal pain, and vaginal bleeding. The similar clinical symptomatology of ovarian pregnancy to ectopic tubal pregnancy makes preoperative diagnosis hardly impossible (Mehmood and Thomas, 1985). When accompanied by a large amount of intra-abdominal hemorrhage, the clinical manifestations of hemorrhagic anemia and hemorrhagic shock can be expressed (Resta et al., 2012). Critical criteria make it difficult to distinguish a ruptured ovarian pregnancy from a fallopian pregnancy rupture, corpus luteum cyst rupture, or ovarian cyst torsion (Shrestha et al., 2012).

Although ovarian pregnancies in the second (Hwang et al., 2020) or even third trimester of pregnancy (Prabhala et al., 2015) have been reported, most ovarian pregnancies are found in the first trimester (Thanasa et al., 2022; Huang et al., 2022). Most of the patients were treated because of abdominal pain or intraperitoneal hemorrhage secondary to ovarian pregnancy rupture. In case 1, since the patient accepted an embryo transfer, serum hCG and intrauterine conditions were monitored over time after transplantation, and the EP was found early. Therefore, the patient did not experience symptoms related to ovarian pregnancy, whereas in case 2, the patient showed typical symptoms, such as menopause and lower abdominal pain.

### Accessory examination

#### Serum hCG

Elevated serum hCG indicates a state of pregnancy. Similar to those of other sites of EP, hCG will continue to rise in ovarian pregnancies if pregnancy-related activity persists, but the value will generally not double within 48 h.

#### Ultrasound examination

Because most ovarian pregnancies are emergency visits due to abdominal pain, and the time for preoperative examination is limited, ultrasound has become the main diagnostic modality for ovarian pregnancy because of its high accessibility. The recommended ultrasound diagnostic criteria for ovarian pregnancy include 1) the presence of a wide echogenic ring with an internal echo lucent area on the ovarian surface, 2) the presence of an ovarian cortex, including a corpus luteum or follicles around the mass, and 3) the echogenicity of the ring being greater than that of the ovary itself (Raziel et al., 2004; Levine, 2007; Comstock et al., 2005).

The diagnosis of ovarian pregnancy is the same as other types of EP. If the yolk sac or embryonic tissue could be detected outside the uterus on ultrasound, it is definitely diagnosed. However, clinically, a yolk sac or embryo was less commonly observed, and the ultrasonic manifestations of ovarian pregnancy are often atypical and ambiguous with pregnancy with the ovarian corpus luteum (Comstock et al., 2005), and preoperative diagnosis have been accurately made only in 11% of cases (Odejinmi et al., 2014). Ovarian pregnancy rupture needs to be distinguished from ovarian corpus luteum rupture because the structure after rupture is more difficult to distinguish on ultrasound. If the patient’s condition is stable, we recommend consulting an experienced sonographer and dynamically reviewing the ultrasound, to improve the diagnostic accuracy.

Although contrast-enhanced ultrasound is useful for the diagnosis and treatment of certain types of ectopic pregnancies, such as cesarean scar pregnancy (Li et al., 2020), its application in ovarian pregnancy has not been documented.

#### MRI examination

Ultrasound is a simple and reproducible auxiliary method for EP. Choi et al. (2011) suggested that the rate of accurate preoperative diagnosis of ovarian pregnancy by transvaginal ultrasound examination was only 18%. Some scholars believe that MRI is also a useful tool for accurately localizing the implantation site, especially when transvaginal ultrasound findings are insufficient or equivocal (Tamai et al., 2007; Takahashi et al., 2013; Köroğlu et al., 2013). An MRI finding indicating an EP would visualize the presence of extrauterine gestation structures that typically appear as a high-intensity mass containing distinct, low-intensity foci on T2-weighted imaging, which indicate hemorrhage. Shingo et al. (2015) successfully demonstrated on MRI a gestation sac structure confined in the ovary that was not visualized by ultrasound.

In case 1, the patient’s vital signs remained stable throughout the preoperative examination period, which allowed enough time for MRI examination. T2 showed mixed signals dominated by a high signal and a small nodule-like low-signal shadow scattered at the edge. Although no typical images of pregnancy tissue, such as yolk sac structure, were obtained, the T2 images further confirmed the results of ultrasonic consultation and provided an imaging basis for further treatment. We believe that for patients with stable vital signs or mild symptoms, preoperative MRI should be performed to confirm diagnosis.

#### CT examination

Computed tomography (CT) examination, particularly enhanced CT examination, is important in the diagnosis of EPs at specific sites, such as hepatic (Hu et al., 2014), omental (Chen et al., 2015), and retroperitoneal pregnancies (Xu et al., 2022). However, its application in detecting ovarian pregnancies is limited. Although a CT scan can help distinguish a ruptured luteal cyst from a ruptured ectopic pregnancy with bleeding in some cases (Liu et al., 2018), CT examination was mostly used to rule out other causes of intraperitoneal hemorrhage in ruptured ovarian pregnancies.

### Treatment

Ovarian pregnancy can be managed through conservative or surgical treatments. Conservative treatment includes expectant treatment and medication (Practice Committee of the American Society for Reproductive Medicine. 2013). The successful treatment of ovarian EP with systemic methotrexate (MTX) using either single- (Di Luigi et al., 2014) or multiple-dose regimens has been described (Kiran et al., 2005). Successful management with transvaginal or laparoscopic injections of MTX directly into the ovarian EP has also been reported (Mittal et al., 2013; Pagidas et al., 2013). Due to the lack of muscle tissue around ovarian pregnancy lesion sand abundant ovarian blood supply, MTX treatment can destroy the villi, but the blood clot formed after embryo sac necrosis increases the volume of the lesion, which may accelerate the rupture and lead to serious intra-abdominal bleeding. Therefore, although drug treatment can be administered in patients with stable vital signs, it should be performed under close monitoring.

Laparoscopy has become the standard in the management of hemodynamically stable patients with ovarian EP (Joseph et al., 2012; Odejinmi et al., 2009). For patients with massive abdominal bleeding who need emergency treatment or those areas with relatively preliminary medical facilities, laparotomy is a suitable option. Resection of the EP and retention of the ovary is a reasonable surgical objective, particularly in patients desiring future fertility. The resection most commonly involves an ovarian wedge resection to remove as little normal ovarian tissue as possible (Nadarajah et al., 2002). In reports on the surgical management of ovarian EP, hemostasis with ultrasonic energy, rather the electrocautery and local injection of argipressin into the border of the normal ovary and the lesion, were used to conserve as much normal ovarian tissue as possible (Eskandar. 2010; Kaur et al., 2019).

For the cases presented herein, we performed laparoscopic surgery. To protect the ovaries as much as possible during the operation, we used laparoscopic scissors to separate and remove the pregnancy lesions and sutured the ovaries with an absorbable suture instead of electrocoagulation hemostasis so as to minimize damage by heat injury to the ovary, and we removed the gestational sac intact to avoid a secondary operation or drug treatment (MTX) caused by residual focus. We placed the suture inside the ovary during the process of suturing and applied anti-adhesion biomaterials on the wound of the ovary to reduce the occurrence of adhesion and protect ovarian function.

### Prognosis

Trophoblastic tissue may persist following conservative surgical management and requires postoperative hCG tracking. The 12 patients presented in case series, conversely, required no further treatment Odejinmi et al. (2009). In the two cases presented here, hCG levels gradually normalized after operation, and there was no fluctuation in the hCG value. Koo et al. (2001) described the long-term outcomes of 28 ovarian pregnancies that were followed up for at least 1 year postoperatively and found no recurrences. Herein, we have conducted follow-ups for 16 and 5 months, respectively. The patients recovered well after the operation, and the patient in case 1 was able to successfully conceive *via* a second embryo transfer.

In conclusion, ovarian pregnancies are rare, and unruptured ovarian pregnancies are rarer. The unruptured cases herein demonstrate the possibility of an unruptured and viable ovarian pregnancy, which can help us better understand the clinical manifestations, imaging findings, and laparoscopic morphology of ovarian pregnancy. Once the ovarian pregnancy is ruptured, a massive hemorrhage can lead to severe complications. Understanding the clinical characteristics of an unruptured ovarian pregnancy can allow early detection and implementation of an active treatment plan to reduce the risk of serious complications. More definitive diagnosis of ovarian pregnancies must be reported in order to better understand the condition and identify optimal preoperative examination methods and management strategies (Goyal et al., 2014; Kiran et al., 2005; Koo et al., 2011).

## Abbreviations

CDFI, color Doppler flow imaging; CT, computed tomography; EP, ectopic pregnancy; hCG, human chorionic gonadotropin; IVF-ET, *in vitro* fertilization and embryo transfer; IUD, intrauterine device; MRI, magnetic resonance imaging; MTX, methotrexate.

## References

[B1] Abidi A. Gordon R. Harman M. B. Pinto M. (2017). Ovarian pregnancy without definitive pathologic confirmation: A case report. J. Reprod. Med. 52, 320 17506374

[B2] Cabero A. Laso E. Lain J. M. Manas C. Escribano I. Calaf J. (1989). Increasing incidence of ovarian pregnancy. Eur. J. Obstet. Gynecol. Reprod. Biol. 31, 227–232. 10.1016/0028-2243(89)90157-3 2787761

[B3] Chen L. Qiu L. Diao X. Yue Q. Gong Q. (2015). CT findings of omental pregnancy: A case report. Jpn. J. Radiol. 33 (8), 499–502. 10.1007/s11604-015-0449-7 26111878

[B4] Choi H. J. Im K. S. Jung H. J. Lim K. T. Mok J. E. Kwon Y. S. (2011). Clinical analysis of ovarian pregnancy: A report of 49 cases. Eur. J. Obstet. Gynecol. Reprod. Biol. 158 (1), 87–89. 10.1016/j.ejogrb.2011.04.015 21601978

[B5] Ciortea R. Costin N. Chiroiu B. Mălutan A. Mocan R. Hudacsko A. (2013). Ovarian pregnancy associated with pelvic adhesions. Clujul Med. 86, 77 26527922 PMC4462474

[B6] Comstock C. Huston K. Lee W. (2004). The ultrasonographic appearance of ovarian ectopic pregnancies. Obstet. Gynecol. 105, 42–45. 10.1097/01.AOG.0000148271.27446.30 15625140

[B7] Di Luigi G. Patacchiola F. La Posta V. Bonitatibus A. Ruggeri G. Carta G. (2012). Early ovarian pregnancy diagnosed by ultrasound and successfully treated with multidose methotrexate. A case report. Clin. Exp. Obstet. Gynecol. 39, 390 23157054

[B8] Dursun P. Gultekin M. Zeyneloglu B. (2008). Ovarian ectopic pregnancy after ICSI-et: A case report and literature review. Arch. Gynecol. Obstet. 278, 191–193. 10.1007/s00404-008-0562-2 18236060

[B9] Einenkel J. Baier D. Horn C. Alexander H. (2000). Laparoscopic therapy of an intact primary ovarian pregnancy with ovarian hyperstimulation syndrome: Case report. Hum. Reprod. 15, 2037–2040. 10.1093/humrep/15.9.2037 10967011

[B10] Eskandar O. (2010). Conservative laparoscopic management of a case of ruptured ovarian ectopic pregnancy by using a Harmonic scalpel. J. Obstet. Gynaecol. 30, 67–69. 10.3109/01443610903295912 20121515

[B11] Ghasemi Tehrani H. Hamoush Z. Ghasemi M. Hashemi L. (2014). Ovarian ectopic pregnancy: A rare case. Iran. J. Reprod. Med. 12, 281 24976824 PMC4071634

[B12] Ghi T. Banfi A. Marconi R. Iaco P. D. Pilu G. Aloysio D. D. (2005). Three-dimensional sonographic diagnosis of ovarian pregnancy. Ultrasound Obstet. Gynecol. 26 (1), 102–104. 10.1002/uog.1933 15971283

[B13] Goyal L. D. Tondon R. Goel P. Sehgal A. (2014). Ovarian ectopic pregnancy: A 10 years' experience and review of literature. Iran. J. Reprod. Med. 12 (12), 825–830. 25709640 PMC4330663

[B14] Herbertsson G. Magnusson S. S. Benediktsdottir K. (1987). Ovarian pregnancy and IUCD use in a defined complete population. Acta Obstet. Gynecol. Scand. 66, 607–610. 10.3109/00016348709022065 3439441

[B15] Hu S. Song Q. Chen K. Chen Y. (2014). Contrast-enhanced multiphasic CT and MRI of primary hepatic pregnancy: A case report and literature review. Abdom. Imaging 9 (4), 731–735. 10.1007/s00261-014-0101-5 24531351

[B16] Huang Y. Huang Q. Liu J. Guo M. Liu Y. Lai D. (2022). Concurrent ovarian and tubal ectopic pregnancy after IVF-et: Case report and literature review. Front. Physiol. 13, 850180. 10.3389/fphys.2022.850180 35444560 PMC9013932

[B17] Hwang D. W. Choi H. W. Choi Y. Y. Kim H. S. Kim Y. A. Chun K. C. (2020). Ovarian pregnancy rupture in second trimester manifesting mental change in pregnancy: A case report. Obstet. Gynecol. Sci. 63 (2), 209–212. 10.5468/ogs.2020.63.2.209 32206662 PMC7073367

[B18] Io S. Hasegawa M. Koyama T. (2015). A case of ovarian pregnancy diagnosed by MRI. Case Rep. Obstet. Gynecol. 2015, 143031. 10.1155/2015/143031 26491583 PMC4600503

[B19] Joseph R. J. Irvine L. M. (2012). Ovarian ectopic pregnancy: Aetiology, diagnosis, and challenges in surgical management. J. Obstet. Gynaecol. 32, 472–474. 10.3109/01443615.2012.673039 22663322

[B20] Kaur N. Reid F. Ma K. (2019). Ovarian ectopic pregnancy: Laparoscopic excision and ovarian conservation. J. Minim. Invasive Gynecol. 26, 1006. 10.1016/j.jmig.2018.12.017 30615953

[B21] Kiran G. Guven A. M. Köstü B. (2005). Systemic medical management of ovarian pregnancy. Int. J. Gynaecol. Obstet. 91, 177–178. 10.1016/j.ijgo.2005.07.010 16168990

[B22] Koo Y. J. Choi H. J. Im K. S. Jung H. J. Kwon Y. S. (2011). Pregnancy outcomes after surgical treatment of ovarian pregnancy. Int. J. Gynaecol. Obstet. 114, 97–100. 10.1016/j.ijgo.2011.02.013 21669418

[B23] Köroğlu M. Kayhan A. Soylu F. N. Erol B. Gurses C. (2013). MR imaging of ectopic pregnancy with an emphasis on unusual implantation sites. Jpn. J. Radiol. 31 (2), 75–80. 10.1007/s11604-012-0151-y 23132557

[B24] Levine D. (2007). Ectopic pregnancy. Radiology 245, 385–397. 10.1148/radiol.2452061031 17940301

[B25] Li H. Liu X. Xie L. Ye Z. Gan L. (2020). Diagnostic accuracy and cut-off of contrast-enhanced ultrasound in caesarean scar pregnancy. Eur. J. Obstet. Gynecol. Reprod. Biol. 246, 117–122. 10.1016/j.ejogrb.2020.01.036 32007793

[B26] Liu X. Song L. Wang J. Liu Q. Liu Y. Zhang X. (2018). Diagnostic utility of CT in differentiating between ruptured ovarian corpus luteal cyst and ruptured ectopic pregnancy with hemorrhage. J. Ovarian Res. 11 (1), 5. 10.1186/s13048-017-0374-8 29316947 PMC5761095

[B27] Marcus S. F. Brinsden P. R. (1993). Primary ovarian pregnancy after *in vitro* fertilization and embryo transfer: Report of seven cases. Fertil. Steril. 60, 167–169. 10.1016/s0015-0282(16)56057-9 8513937

[B28] Mehmood S. A. Thomas J. A. (1985). Primary ectopic ovarian pregnancy (report of three cases). J. Postgrad. Med. 31 (4), 219 3834088

[B29] Mittal S. Dadhwal V. Baurasi P. (2013). Successful medical management of ovarian pregnancy. Int. J. Gynaecol. Obstet. 80, 309–310. 10.1016/s0020-7292(02)00304-1 12628534

[B30] Nadarajah S. Sim L. N. Lo S. F. (2002). Laparoscopic management of an ovarian pregnancy. Singap. Med. J. 43, 095 11993898

[B31] Nakagawa K. Enari T. Kaneko E. Kawamura H. Santou A. Uesato T. (20042004). Ovarian pregnancy: Report of 13 cases in recent ten years. Jpn. J. Gynecol. Obstet. Endosc. 20, 158. 10.5180/jsgoe.20.2_158

[B32] Odejinmi F. Rizzuto M. I. MacRae R. Olowu O. Hussain M. (2009). Diagnosis and laparoscopic management of 12 consecutive cases of ovarian pregnancy and review of literature. J. Minim. Invasive Gynecol. 16 (3), 354–359. 10.1016/j.jmig.2009.01.002 19423068

[B33] Pagidas K. Frishman G. N. (2013). Nonsurgical management of primary ovarian pregnancy with transvaginal ultrasound-guided local administration of methotrexate. J. Minim. Invasive Gynecol. 20, 252–254. 10.1016/j.jmig.2012.10.012 23465263

[B34] Paltieli Y. Eibschitz I. Ziskind G. Ohel G. Silbermann M. Weichselbaum A. (2000). High progesterone levels and ciliary dysfunction-a possible cause of ectopic pregnancy. J. Assist. Reprod. Genet. 17 (2), 103–106. 10.1023/a:1009465900824 10806589 PMC3455158

[B35] Prabhala S. Erukkambattu J. Dogiparthi A. Kumar P. Tanikella R. (2015). Ruptured ovarian pregnancy in a primigravida. Int. J. Appl. Basic Med. Res. 5, 151–153. 10.4103/2229-516X.157175 26097828 PMC4456894

[B36] Practice Committee of the American Society for Reproductive Medicine (2013). Medical treatment of ectopic pregnancy: A committee opinion. Fertil. Steril. 100, 638–644. 10.1016/j.fertnstert.2013.06.013 23849842

[B37] Raziel A. Golan A. Pansky M. Ron-El R. Bukovsky I. Caspi E. (1990). Ovarian pregnancy: A report of twenty cases in one institution. Am. J. Obstet. Gynecol. 163, 1182–1185. 10.1016/0002-9378(90)90685-z 2220925

[B38] Raziel A. Schachter M. Mordechai E. Friedler S. Panski M. Ron-El R. (2004). Ovarian pregnancy-a 12-year experience of 19 cases in one institution. Eur. J. Obstet. Gynecol. Reprod. Biol. 114, 92–96. 10.1016/j.ejogrb.2003.09.038 15099878

[B39] Resta S. Fuggetta E. D'Itri F. Evangelista S. Ticino A. Porpora M. G. (20122012). Rupture of ovarian pregnancy in a woman with low beta-hCG levels. Case Rep. Obstet. Gynecol. 2012, 213169. 10.1155/2012/213169 PMC3502786 23198195

[B40] Rizk B. Tan S. L. Morcos S. Riddle A. Brinsden P. Mason B. A. (1991). Heterotopic pregnancies after *in vitro* fertilization and embryo transfer. Am. J. Obstet. Gynecol. 164 (1 Pt 1), 161–164. 10.1016/0002-9378(91)90648-b 1986604

[B41] Selo-Ojeme O. Goodfellow F. (2002). Simultaneous intrauterine and ovarian pregnancy following treatment with clomiphene citrate. Arch. Gynecol. Obstet. 266, 232–234. 10.1007/s004040100213 12192486

[B42] Sergent F. Mauger-Tinlot F. Gravier A. Verspyck E. (2002). Ovarian pregnancies: Revaluation of diagnostic criteria. J. Gynecol. Obstet. Biol. Reprod. 31, 741 12592193

[B43] Shrestha A. Chawla C. D. Shrestha R. M. (2012). Ruptured primary ovarian pregnancy: A rare case report. Kathmandu Univ. Med. J. 10 (39), 76–77. 10.3126/kumj.v10i3.8026 23434969

[B44] Spiegelberg O. (1973). Zur Cosuistik der ovarialschwanger schalt. Arch. Gynak. 13, 73–79. 10.1007/bf01991416

[B45] Suikkari A. M. Söderström-Anttila V. (2007). *In-vitro* maturation of eggs: Is it really useful? Best. Pract. Res. Clin. Obstet. Gynaecol. 21 (1), 145–155. 10.1016/j.bpobgyn.2006.09.003 17291833

[B46] Takahashi A. Takahama J. Marugami N. Takewa M. Itoh T. Kitano S. (2013). Ectopic pregnancy: MRI findings and clinical utility. Abdom. Imaging 38 (4), 844–850. 10.1007/s00261-012-9969-0 23161059

[B47] Tamai K. Koyama T. Togashi K. (2007). MR features of ectopic pregnancy. Eur. Radiol. 17 (12), 3236–3246. 10.1007/s00330-007-0751-6 17882426

[B48] Tan K. K. Yeo O. H. (1968). Primary ovarian pregnancy. Am. J. Obstet. Gynecol. 100, 240–249. 10.1016/s0002-9378(15)33727-3

[B49] Thanasa E. Thanasa A. Gerokostas E. E. Kamaretsos E. Koutalia N. Kontogeorgis G. (2022). Rupture of ectopic ovarian pregnancy accompanied by massive intra-abdominal bleeding and disorder of the coagulation mechanism: A rare and life-threatening obstetric complication. Cureus 14 (8), e28112. 10.7759/cureus.28112 36127987 PMC9481053

[B50] Xu H. Cheng D. Yang Q. Wang D. (2022). Multidisciplinary treatment of retroperitoneal ectopic pregnancy: A case report and literature review. BMC Pregnancy Childbirth 22 (1), 472. 10.1186/s12884-022-04799-5 35672717 PMC9175374

[B51] Zhu Q. Li C. Zhao W. H. Yuan J. J. Yan M. X. Qin G. J. (2014). Risk factors and clinical features of ovarian pregnancy: A case-control study. BMJ Open 4 (12), e006447. 10.1136/bmjopen-2014-006447 PMC4256641 25472658

